# Evaluation of the Reliability of AI-Based Large Language Models in Developing Orthodontic Treatment Plans

**DOI:** 10.7759/cureus.89149

**Published:** 2025-07-31

**Authors:** Makara Sorel, Chaitanya Gurrala, Aditya Tadinada

**Affiliations:** 1 Oral and Maxillofacial Radiology, University of Connecticut (UConn) School of Dental Medicine, Farmington, USA; 2 Orthodontics and Dentofacial Orthopedics, University of Connecticut (UConn) School of Dental Medicine, Farmington, USA

**Keywords:** artificial intelligence, artificial intelligence in dentistry, artificial intelligence in healthcare, chatgpt, google gemini, orthodontics, orthodontic treatment plan

## Abstract

Background and aim

Orthodontic treatment planning is a complex process requiring a detailed understanding of dental, skeletal, and soft tissue relationships. Traditionally, treatment decisions are made through clinical expertise and evidence-based guidelines. However, the recent evolution of AI, particularly large language models (LLMs), has warranted an evaluation of their capabilities in streamlining clinical workflows. The aim of this study was to evaluate the proficiency and effectiveness of AI-based LLMs, specifically OpenAI’s ChatGPT-4o and Google’s Gemini 2.0 Flash Experimental (free version), in generating orthodontic treatment plans based on real clinical cases.

Materials and methods

Ten published orthodontic case reports from reputed peer-reviewed journals were selected for the study and summarized into standardized clinical inputs, including patient age, occlusal relationships, skeletal and dental findings, and radiographic observations. These inputs were submitted to ChatGPT-4o and Gemini 2.0 Flash Experimental (free version) with prompts to generate extremely detailed, comprehensive treatment plans. The outputs were evaluated independently by two experienced orthodontists and one orthodontic resident using a four-point ordinal scale assessing clinical accuracy, completeness, and relevance of the treatment plan. Inter-rater reliability was assessed using Krippendorff’s alpha.

Results

ChatGPT-4o produced treatment plans with higher clinical alignment and evaluator consensus, as indicated by Krippendorff’s alpha (α = 0.935), while Gemini’s plans showed greater variability and moderate agreement (α = 0.692). ChatGPT generated orthodontic treatment plans that incorporated more relevant clinical details and demonstrated stronger alignment with evidence-based standards, as assessed by the orthodontic reviewers. In contrast, Gemini generated treatment plans based on minimally accurate facts.

Conclusion

LLMs such as ChatGPT-4o and Gemini 2.0 Flash Experimental (free version) demonstrate potential as valuable complementary tools in orthodontic treatment planning, especially in routine cases, but do not appear to have the ability to replace clinical expertise.

## Introduction

Orthodontics primarily focuses on the diagnosis of malocclusion and the development of structured treatment plans. Malocclusion can negatively impact occlusal function and cause psychological distress, ultimately diminishing quality of life [1]. The main objective of orthodontic treatment is to address these issues by maximizing patient benefits while minimizing potential risks. Treatment plans are based on clinical findings and diagnostic test results and may vary depending on individual cases.

AI has advanced significantly, streamlining tasks and augmenting human capabilities. Commercialized large language models (LLMs), such as OpenAI’s ChatGPT and Google AI’s Gemini, are emerging as powerful problem-solving tools across industries [2]. OpenAI’s GPT-4o, released in May 2024, processes and responds to text, audio, and images, leveraging Reinforcement Learning with Human Feedback (RLHF) for improved accuracy. It was trained on diverse datasets, including public web data, code, and multimodal inputs [3].

Similarly, Google’s Gemini 2.0 Flash Thinking Experimental (free version), formerly Bard, is designed for enhanced reasoning and excels in math, science, and long-form text analysis [4]. Unlike ChatGPT, at the time this study was conducted, Gemini lacked audio input processing and non-text output generation [5]. In addition, both products have access to the internet for real-time web searches.

Despite AI’s rapid progress, LLMs have yet to be widely applied in orthodontics, particularly in treatment planning, a critical aspect of patient care. AI’s ability to generate orthodontic treatment plans remains largely unexplored, though its role in digital workflows has already improved diagnostic accuracy and efficiency [6].

There is a clear need for a novel study like this to assess the current capability of LLMs, specifically Gemini and ChatGPT-4o, in generating accurate orthodontic treatment plans. Given that orthodontic treatment directly impacts patient health and well-being, treatment plans must be comprehensive, precise, and tailored to each patient’s unique needs.

Objective

The objective of this study is to evaluate the proficiency and effectiveness of ChatGPT-4o and Gemini 2.0 Flash Thinking Experimental (free version) in developing orthodontic treatment plans. Through this evaluation, the study seeks to determine the potential role of AI in orthodontic treatment planning and its implications for the future of digital orthodontics.

This research was previously presented as a poster at the Connecticut State Dental Association on May 16, 2024.

## Materials and methods

A total of 10 published orthodontic case reports from orthodontic journals were selected for this study [7-16]. These case reports were retrieved through the UConn Health Library databases. The literature for this paper was identified and selected by performing a thorough search in UConn Health Library databases such as PubMed, Google Scholar, Medline, Embase, and Cochrane, focusing on studies published over the past decade. The selected cases included patients who had a full set of orthodontic records, including panoramic images, lateral cephalometric radiographs, intraoral scans, and extraoral and intraoral photographs (frontal, smile, profile, right lateral, left lateral, and occlusal). Of the 10 patients, four were male and six were female, with an average age of 12 years.

**Figure 1:**
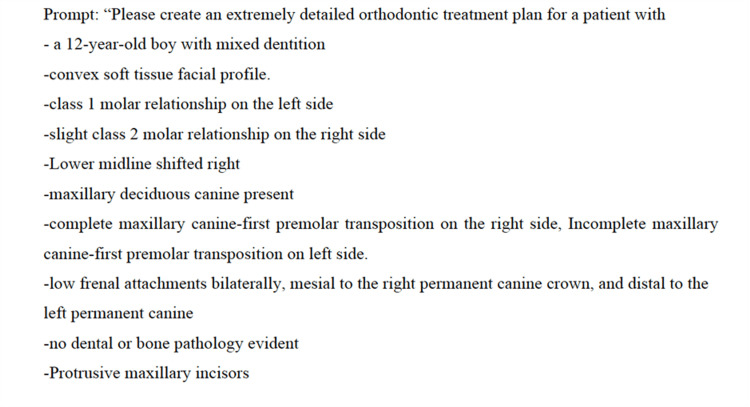
Prompt with elaborate details of patient orthodontic records. This figure shows one of the standardized input prompts derived from published orthodontic case reports and used in the study. It includes detailed clinical findings for a 12-year-old patient with mixed dentition. The prompt was presented to both ChatGPT and Gemini to evaluate their ability to generate accurate orthodontic treatment plans based on real-world diagnostic information.

The orthodontic records of these 10 patients, including panoramic images, intraoral scans, and extraoral and intraoral photographs, were summarized in written format. The LLMs used in this study were: ChatGPT model GPT-4o (free version) and Gemini 2.0 Flash Thinking Experimental (free version). The summarized details were then input into both ChatGPT and Gemini with a prompt to generate a detailed orthodontic treatment plan suitable for implementation (Figure 1). The input texts, which included accurate and elaborate descriptions of the patients' soft and hard tissue characteristics, were reviewed and agreed upon by the authors before submission to the language models. Each input was provided to the LLMs by one author, with no follow-up questions, rephrasing, or additional explanations after the initial prompt was given.

The treatment plans generated by ChatGPT and Gemini were evaluated and scored by an orthodontic resident and two experienced orthodontists. A specific scale ranging from one to four, one being the highest rating and four the lowest, was used to assess the accuracy and utility of the treatment plans (Table 1). Statistical analysis using Krippendorff's alpha was performed to measure the inter-rater agreement among the three evaluators.

**Table 1:** Scoring table used to evaluate AI-generated orthodontic treatment plans. This table outlines a four-point scale used by two orthodontists and one orthodontic resident to assess the accuracy and utility of treatment plans produced by ChatGPT and Gemini. Scores range from 1 (Objectively true), indicating comprehensive and evidence-based plans, to 4 (False), indicating plans lacking evidence or containing contradictory information.

Category	Definition
Objectively true (1)	Treatment plan based on available scientific evidence and includes all relevant information necessary for treatment.
Selected facts (2)	Treatment plan based on selected facts and scientific evidence but omits important information related to the treatment plan.
Minimal facts (3)	Treatment plan that presents only minimally accurate facts.
False (4)	Treatment plan that is objectively false due to lack of supporting evidence or contradiction of available evidence.

The intent was to evaluate the ability of two LLMs, ChatGPT and Gemini, to independently generate usable orthodontic treatment plans and then assess and compare the responses provided by the two models based on the same clinical inputs.

## Results

**Figure 2:**
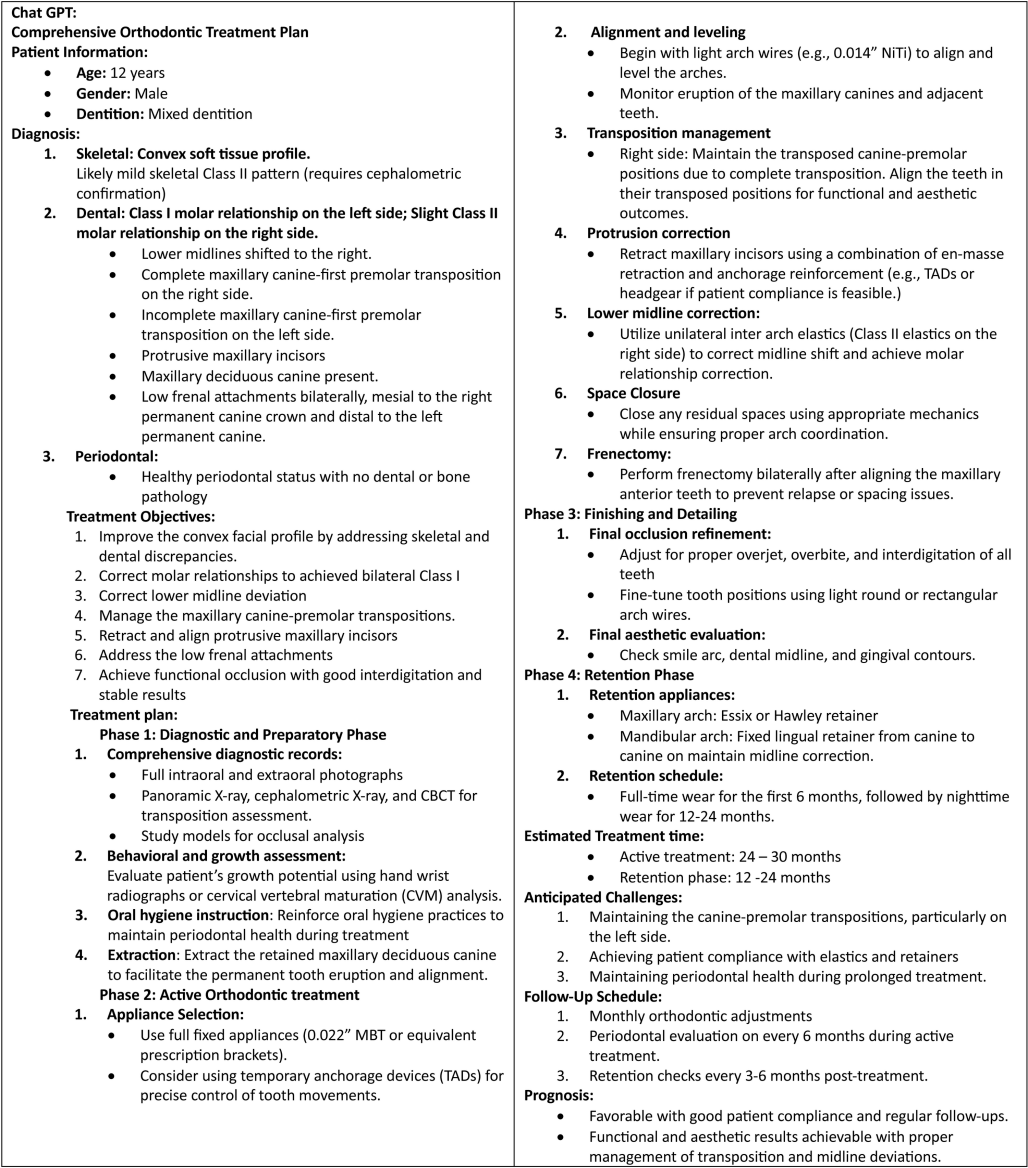
Comprehensive treatment plan generated by ChatGPT. Structured orthodontic treatment plan created by ChatGPT, outlining the diagnostic summary, treatment objectives, and phased intervention strategy (diagnostic, active treatment, detailing, and retention) for a 12-year-old orthodontic patient. The response shown is from January 19, 2025.

**Figure 3:**
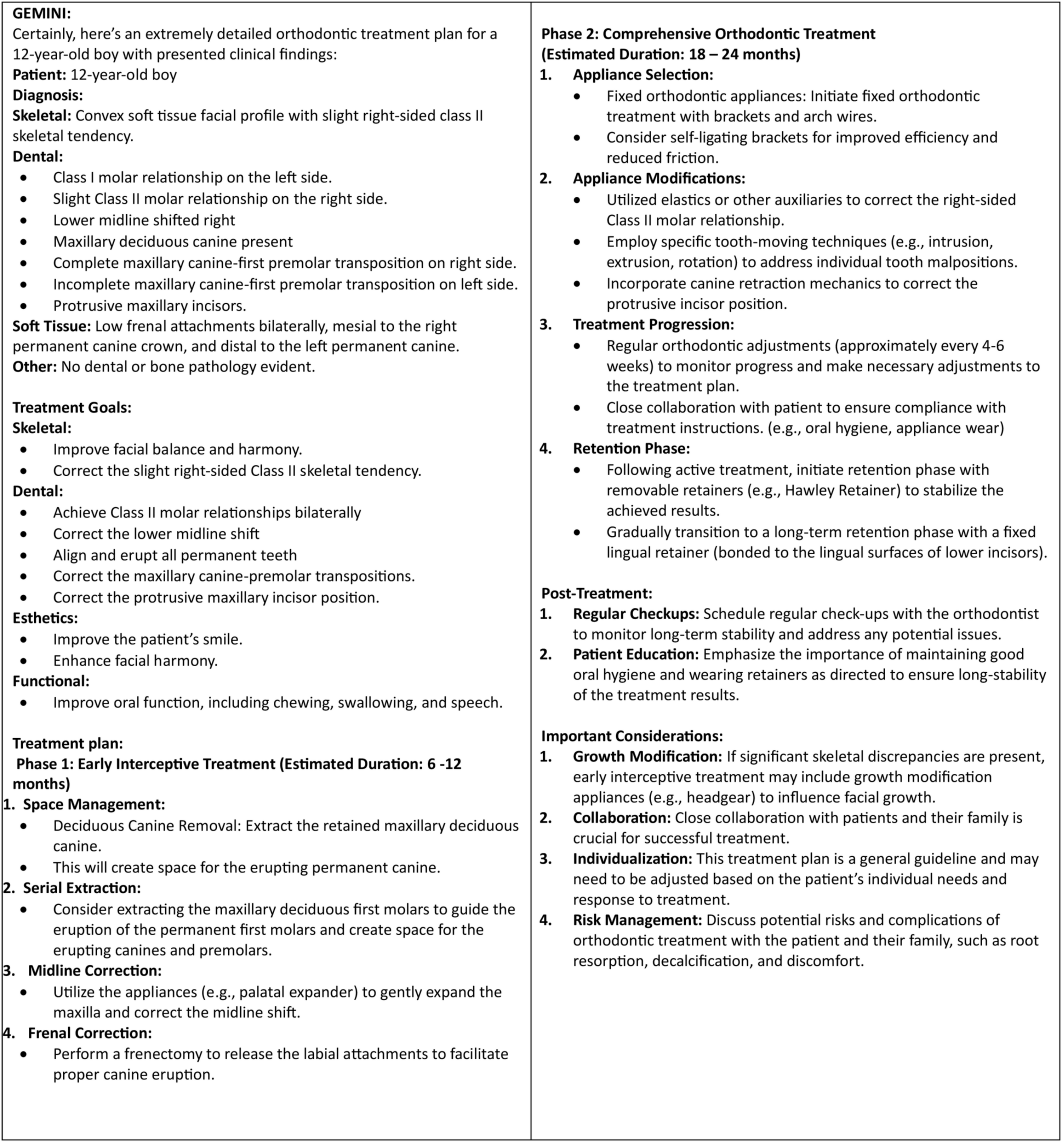
Comprehensive treatment plan generated by Gemini. This figure displays a structured orthodontic treatment plan created by Gemini for a 12-year-old orthodontic patient. The response shown is from January 19, 2025.

The treatment plans generated by both AI platforms, ChatGPT (Figure 2) and Gemini (Figure 3), were evaluated and scored independently by three experienced orthodontists (Tables 2-3). Each plan was scored on a 4-point ordinal scale (one to four) based on its scientific accuracy, completeness, and clinical relevance. The inter-rater agreement among the three orthodontic evaluators was assessed using Krippendorff’s alpha for both AI platforms. To measure the inter-rater agreement, Krippendorff’s alpha (α) was calculated using the following formula and Python software.

$\alpha=1-\frac{D_{o} }{D_{e}}$

Where: 

· Dₒ = Observed disagreement among raters

· Dₑ = Expected disagreement by chance

**Table 2:** Scoring data for ChatGPT as evaluated by examiners. Each case was independently evaluated by three examiners using the 4-point scale previously described, where 1 = objectively true, 2 = selected facts, 3 = minimal facts, and 4 = false. The table shows consistent scoring across most cases, with minor variation observed in Case 8.

Case No.	Examiner 1	Examiner 2	Examiner 3
1	2	2	2
2	2	2	2
3	2	2	2
4	2	2	2
5	2	2	2
6	2	2	2
7	2	2	2
8	2	3	3
9	2	2	2
10	2	2	2

**Table 3:** Scoring data for Gemini as evaluated by examiners. Each case was independently evaluated by three examiners using the 4-point scale previously described: 1 = Objectively true, 2 = Selected facts, 3 = Minimal facts, 4 = False. The table shows largely consistent scoring across all cases, with slight variation observed in Cases 2, 6, and 9.

Case No.	Examiner 1	Examiner 2	Examiner 3
1	3	3	3
2	3	2	3
3	3	3	3
4	3	3	3
5	3	3	3
6	3	2	2
7	3	3	3
8	3	3	3
9	3	3	2
10	3	3	3

**Figure 4:**
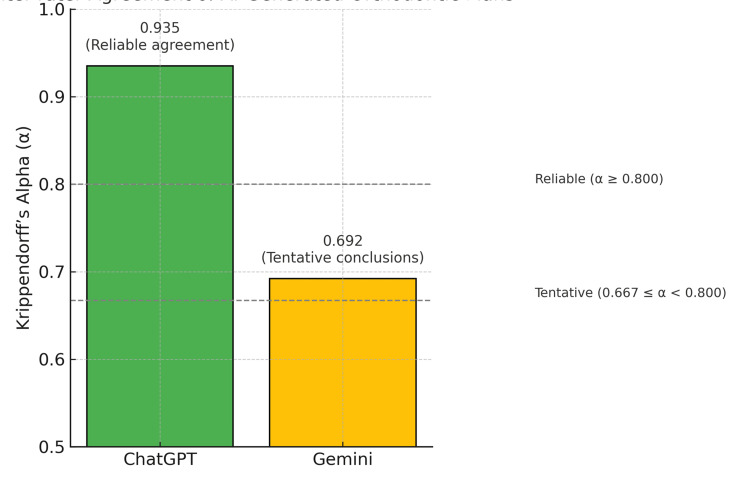
Inter-rater agreement (Krippendorff’s alpha) for ChatGPT and Gemini. The green bar represents ChatGPT (α = 0.935), indicating reliable agreement. The yellow bar represents Gemini (α = 0.692), suggesting tentative conclusions. Dashed lines mark interpretation thresholds at α = 0.800 (reliable agreement) and α = 0.667 (minimum acceptable agreement).

ChatGPT demonstrated a high level of agreement with a Krippendorff’s alpha value of 0.935, indicating reliable consistency among evaluators. In contrast, Gemini yielded a Krippendorff’s alpha of 0.692, reflecting moderate agreement based on Krippendorff’s interpretive scale (Table 4 and Figure 4).

**Table 4:** Inter-rater agreement using Krippendorff’s alpha. Krippendorff’s alpha (𝛼) was used to assess inter-rater reliability among evaluators for both ChatGPT and Gemini. Values closer to 1.0 indicate higher agreement. ChatGPT demonstrated high agreement (𝛼 = 0.935), while Gemini showed moderate agreement (𝛼 = 0.692).

AI Platform	Krippendorff’s Alpha (𝛼)	Interpretation
ChatGPT	0.935	High agreement
Gemini	0.692	Moderate agreement

These findings suggest that ChatGPT generated orthodontic treatment plans with greater inclusion of selected facts and stronger alignment with evidence-based standards, as perceived by the orthodontic reviewers, compared to Gemini, which generated treatment plans based on minimally accurate facts.

## Discussion

The integration of computers has transformed healthcare, and the introduction of AI has further enhanced this impact. AI-driven systems use computational models capable of rational thinking and decision-making, acting as valuable support tools for healthcare professionals. By increasing efficiency and saving time, AI improves patient care and optimizes clinical workflows. In recent years, AI has been used in orthodontics to identify cephalometric landmarks [17,18], determine the need for orthodontic extractions [19,20], assess the degree of cervical vertebra maturation [21], predict facial attractiveness after orthognathic surgery [22], and assist in treatment planning [23].

However, no literature currently exists on using AI to create a comprehensive treatment plan tailored to each individual patient. This study examines the scope and effectiveness of two distinct AI-based models (ChatGPT and Gemini) in different aspects of orthodontic diagnosis and treatment planning for ten patients.

Shimizu Y et al. [24] conducted a study to validate AI systems in diagnosis and treatment planning. They represented patients’ clinical information as a vectorized bag of words to develop a treatment plan that four orthodontists evaluated. They concluded that AI performed significantly worse in treatment planning. In contrast to their approach, we summarized each patient’s clinical features in detail and submitted these summaries to ChatGPT and Gemini, prompting the models to generate detailed treatment plans.

Makrygiannakis MA et al. [25] investigated the evidence-based capabilities of four LLMs, ChatGPT-3.5, ChatGPT-4, Google Bard, and Microsoft Bing, within orthodontics. They provided each model with ten open-ended clinical questions and had two orthodontists assess the responses using a 0 to 10 rating scale. The results revealed that Microsoft Bing Chat attained the highest score, followed by ChatGPT-4, Google Bard, and then ChatGPT-3.5. Although we did not include Microsoft Bing Chat in our study, ChatGPT-4 received a higher score than Gemini. The treatment plans generated by ChatGPT were consistently rated higher by the three evaluators.

Differences in network architectures and variations in the quantity and diversity of training data contribute to LLMs producing distinct and varied responses to identical queries, resulting in different strengths, weaknesses, capabilities, and limitations. This variation arises from the unique frameworks upon which these models are developed. For example, ChatGPT is built using the GPT (Generative Pre-trained Transformer) architecture, which utilizes deep learning through extensive pre-training on large datasets, followed by fine-tuning and alignment techniques such as Reinforcement Learning from Human Feedback (RLHF) [26]. On the other hand, Google Gemini, which replaced Bard, is based on a multimodal architecture developed by Google. Unlike Bard's original LaMDA foundation, Gemini is designed to process and integrate multiple types of input (e.g., text, code, images), with an emphasis on contextual understanding and advanced reasoning capabilities [4].

Tanaka OM et al. [27] analyzed ChatGPT’s effectiveness in delivering accurate and high-quality responses to queries regarding clear aligners and temporary anchorage devices. Their study determined that ChatGPT is capable of producing reliable information on these topics, as well as on digital imaging within orthodontics. However, only ten questions were posed to the AI system, which may not comprehensively represent the entirety of orthodontic knowledge.

In our study, we found that using AI for diagnosis and treatment planning offers both advantages and limitations in orthodontics. On the one hand, AI-assisted diagnosis and planning could allow orthodontists to spend more time discussing treatment options with patients, as the AI can propose multiple alternatives. This may help reduce the risk of overlooking key aspects of a patient’s individual condition. On the other hand, AI should be used cautiously in diagnosis and treatment planning. The final responsibility for clinical decisions made with support from AI systems must rest with the orthodontist.

Limitations

A key limitation of AI is its dependence on past data, which can pose challenges when encountering novel or complex cases. In this study, when confronted with intricate situations such as canine impaction or missing premolars, the AI systems often provided generic suggestions like “correct the canine impaction,” without offering a comprehensive treatment strategy. Although AI can improve orthodontic workflow efficiency, it currently lacks the reliability needed for autonomous clinical decision-making and still requires expert oversight. While it can support less experienced orthodontists, it should be used strictly as a decision-support tool rather than a replacement for clinical expertise.

Several limitations associated with this study also warrant mention. First, we used only 10 randomly selected case reports, which may not reflect the full spectrum of orthodontic presentations. Second, the actual implementation and clinical outcomes of the AI-generated treatment plans were not evaluated. Third, the AI tools used in this study are continuously evolving, and their diagnostic capabilities may improve over time, potentially altering how they respond to similar clinical scenarios in the future.

## Conclusions

LLMs, such as ChatGPT-4o and Gemini 2.0 Flash Thinking Experimental (free version), show promise as complementary tools in orthodontic treatment planning. Although they are not a substitute for clinical expertise, these models can assist practitioners by providing preliminary treatment suggestions, particularly in routine cases. However, their limitations in addressing complex clinical scenarios highlight the continued need for expert oversight. At present, LLMs should serve as supportive aids, not replacements, for orthodontic decision-making.
